# Immune Checkpoint Blockade in Hematological Malignancies: Current Status and Future Directions

**DOI:** 10.3390/cancers18030485

**Published:** 2026-01-31

**Authors:** Hiu-Ching Lau, Yok-Lam Kwong

**Affiliations:** Department of Medicine, Queen Mary Hospital, Hong Kong, China

**Keywords:** immune checkpoint inhibitor, classical Hodgkin lymphoma, primary mediastinal large B-cell lymphoma, NK/T-cell lymphoma

## Abstract

Immune checkpoint proteins including PD-1, CTLA-4, LAG-3, TIM-3, and TIGIT regulate T-cell functions, which are essential for anti-tumor immunity. Over-expression of these immune checkpoint proteins leads to T-cell exhaustion and a significant impairment of anti-tumor immunity. Rejuvenation of effector T-cell function with immune checkpoint inhibitors (ICI) restores anti-tumor immunity, which translates into clinical efficacy in the frontline and salvage treatment of various hematological malignancies. Efficacy of ICIs is highest in classical Hodgkin lymphoma, primary mediastinal large B-cell lymphoma, and NK/T-cell lymphomas, and modest in immune-privileged-site lymphomas and cutaneous T-cell lymphoma. However, in myeloid malignancies and multiple myeloma, the efficacy of ICIs remains doubtful. In addition to being used as single agents, ICIs have also been combined with other ICIs; as well as chemotherapy, antibody drug conjugates, and epigenetic agents (histone deacetylase inhibitors and hypomethylating agents). More innovative strategies include the use of ICIs in the context of allogeneic haematopoietic stem cell transplantation and chimeric antigen receptor T-cell therapy. This review synthesizes current evidence for the use of ICI in different haematological malignancies, and highlights future directions toward biomarker-driven, rationally designed therapeutic combinations.

## Simple Summary

Immune checkpoint inhibitors (ICI) are important options in the treatment of cancers. In hematological malignancies, classical Hodgkin lymphoma, primary mediastinal B-cell lymphoma, NK/T-cell lymphoma, immune-privileged site large B-cell lymphoma, and cutaneous T-cell lymphomas have been shown to be sensitive to ICI in variable proportions. The combination of ICI with conventional chemotherapy and other targeting molecules holds promise in further increasing efficacy and extending the disease spectrum in the management of hematological malignancies.

## 1. Introduction

Immune checkpoint inhibitors (ICIs) have revolutionized the treatment of numerous malignancies since their initial approval in melanoma [1]. By rejuvenating exhausted immune cells, ICIs aim at restoring effective anti-tumor immunity. This approach has shown promising to sometimes spectacular results for various hematological malignancies, including Hodgkin lymphoma [2,3,4], mediastinal large B cell lymphoma [5,6], NK/T-cell lymphoma, immune-privileged site lymphomas and cutaneous T cell lymphomas. ICIs had also been tested in myeloma and myeloid malignancies, although results were modest at best to negative.

This review will provide an overview of the current status of ICIs in hematological malignancies, and insights into potential future directions in the field, including combination approaches with different immunologic therapies, chemotherapeutic drugs, and biological agents.

## 2. T-Cell Activation and Anergy/Exhaustion

T-cell activation is triggered by stimuli such as infection, inflammation, or malignancy. This process begins when antigen-presenting cells (APCs) display pathogenetic antigens bound to major histocompatibility complex (MHC) molecules, which are recognized by T-cell receptors (TCRs). The binding of TCRs to their target antigens delivers the first activation signal. An additional co-stimulation step is required for full T cell activation and proliferation. The prototype T-cell co-stimulatory receptor is CD28. The binding of CD28 on T cells to its ligands CD80 (B7-1) and CD86 (B7-2) on APCs leads to its activation, which initiates a series of signaling cascades mediated via tyrosine phosphorylation. Conversely, T-cells express inhibitory receptors, including CD279 (PD-1), CD223 (LAG3) and CD366 (TIM3), which on ligation with their cognate receptors result in inhibition of T-cell activation.

T-cell activation can be affected by a myriad of reasons. In the absence of CD28 signaling, naïve T lymphocytes fall into a state of unresponsiveness known as anergy [7]. Chronic antigenic stimulation caused by infections or malignancies results in a state of T-cell exhaustion, characterized by impaired effector function and over-expression of inhibitory receptors. Recently, it has been proposed that T-cell exhaustion may be caused by a proteotoxic stress response, mediated by persistent AKT signaling, which leads to disruption of proteostasis, accumulation of protein aggregates and stress granules, and autophagy-dominant protein catabolism; thereby resulting in the exhaustion phenotype [8].

## 3. Suppression of Immune Cell Function and an Immunosuppressive Microenvironment

### 3.1. Cytotoxic T-Lymphocyte-Associated Antigen 4 (CTLA-4)

CTLA-4 is an inhibitory immune checkpoint highly expressed on activated T cells and regulatory T cells (Tregs) [9]. It competes with CD28, but with a much higher ligand affinity, for binding to B7-1 and B7-2 on APCs. Therefore, CTLA-4 expression substantially attenuates CD28-driven T-cell activation. Apart from directly competing with CD28 for its ligands, CTLA-4 also downregulates CD80 and CD86 expression on APCs, thereby limiting the ability of APCs to stimulate T cells via CD28 [10]. Finally, when T-cells are activated, CTLA-4 expression is upregulated, which serves to restrain uncontrolled expansion of activated T cells.

In T-cell leukemias and lymphomas, including a subset of patients with peripheral T-cell lymphoma, mycosis fungoides, Sézary syndrome, angioimmunoblastic T-cell lymphoma, extranodal NK/T-cell lymphoma, and adult T-cell leukemia/lymphoma [11], recurrent activating mutations in CD28 and CD28-CTLA-4 fusion proteins have been identified, which result in increased ligand affinity and amplified costimulatory signals, both promoting tumor immune escape [2].

Anti-CTLA4 monoclonal antibodies, including ipilimumab and tremelimumab, block CTLA-4, hence freeing CD80/CD86 for binding to CD28 to re-activate its signaling, thereby restoring T-cell effector function and thus anti-tumor immunity.

### 3.2. Programmed Cell Death Protein 1 (PD-1) and Its Ligands (PD-L1/PD-L2)

PD-1 is a member of the B7-CD28 superfamily, and plays a role analogous to CTLA-4. It is expressed on activated T cells (CD8+ and CD4+), B cells, natural killer T cells and APCs. Unlike CTLA-4, which regulates early T cell activation in lymphoid tissues, the PD-1 axis is crucial for sustained T cell activation. Binding to its ligands programmed cell death protein 1 ligand 1(PD-L1) or PD-L2 recruits SHP-1 and SHP-2 phosphatases, resulting in dephosphorylation of key molecules involved in TCR signaling. This leads to T-cell exhaustion, with reduced T-cell proliferation, survival, and effector functions including cytotoxicity and cytokine release [9].

Tumor cells often inappropriately express PD-L1/PD-L2 to exploit this inhibitory pathway, enabling them to evade immune surveillance and destruction. PD-L1 overexpression may be due to intrinsic and adaptive mechanisms [12]. Intrinsic mechanisms involve cellular or genetic alterations. Activation of intracellular signaling pathways, particularly AKT and STAT, has been shown to upregulate PD-L1 transcription and protein levels in malignant cells. In primary mediastinal B-cell lymphoma, chromosomal rearrangements fusing the *CIITA* transactivator gene with the *PD-L1* or *PD-L2* genes drive constitutive overexpression of these immune checkpoint ligands [13]. Similarly, amplification of chromosome 9p24.1, where the genes for *PD-L1* and *PD-L2* are located, is a recurrent genetic abnormality in classical Hodgkin lymphoma (cHL) that leads to high PD-L1/PD-L2 expression [14]. In extranodal NK/T-cell lymphomas and aggressive NK/T-cell leukemias, Epstein–Barr virus (EBV) infection induces PD-L1 expression through viral latent membrane protein-1 (LMP1)-mediated NF-κB activation. Genomic abnormalities involving the 3′UTR of *PD-L1*/*PD-L2* have also been described [15]. Adaptive mechanisms involve upregulation of PD-L1 expression in the tumor microenvironment. Peripheral blood mononuclear cells within the cutaneous T cell lymphoma (CTCL) tumor microenvironment have been shown to have increased PD-L1 expression upon exposure to interferon-γ (IFN-γ) and interleukin-6 [16].

Anti-PD-1 antibodies (e.g., nivolumab, pembrolizumab, tislelizumab) and anti-PD-L1 antibodies (atezolizumab, avelumab, durvalumab) work by blocking the interaction of PD-1 with PD-L1, thereby releasing T cells from checkpoint inhibition, enhancing their anti-tumor response.

### 3.3. LAG-3

Lymphocyte activation gene-3 (LAG-3) is another immune checkpoint. It is an inhibitory transmembrane protein expressed on multiple cell types including CD4+, CD8+ T cells and Tregs, NK cells, activated B cells, and plasmacytoid dendritic cells. LAG-3 shares significant homology with CD4. It binds to MHC class II molecules, its primary ligand, with an affinity higher than that of CD4, thereby suppressing the proliferation, activation and effector functions of CD4+ and CD8+ T lymphocytes [2]. In persistent antigen stimulation caused by chronic infection or malignancy, enhanced LAG-3 expression on T-cells has been observed in combination with other inhibitory receptors including PD-1, T cell immunoglobulin and ITIM domain (TIGIT), and T cell immunoglobulin-3 (TIM3), which leads to T-cell dysfunction [17]. There have been phase I/II trials investigating the safety and tolerability of anti-LAG3 antibodies in lymphomas [18], as well as the potential of a combination of anti-LAG3 antibodies with anti-PD1 [19]. The majority of clinical trials exploring ICIs that target LAG-3 in hematological malignancies are still ongoing. Studies specifically targeting TIGIT and TIM3 in hematological malignancies with successful or promising results are yet to be reported.

### 3.4. CD47

CD47 is a membrane protein first discovered on the surface of erythrocytes. Senescent erythrocytes are removed from the circulation by macrophages in the spleen and liver. Normal erythrocytes prevent this elimination by expression of CD47, which binds to the macrophage inhibitory receptor signal regulatory protein alpha (SIRPα) [20]. This leads to activation of SHP-1 and SHP-2 phosphatases, inhibiting phagocytes. CD47 is commonly referred to as the “do not eat me” signal. It is commonly over-expressed in lymphomas and myeloid malignancies as a macrophage checkpoint. Magrolimab, an anti-CD47 antibody, has been shown to be effective and tolerated when combined with azacitidine in acute myeloid leukemia (AML) and myelodysplastic neoplasm (MDS) [21], and in combination with rituximab in patients with relapsed/refractory (R/R) diffuse large B cell lymphoma (DLBCL) [22]. Other therapies targeting the CD47-SIRPα pathway, including antibodies, inhibitory peptides, and miRNAs, have shown promise in the treatment of hematological malignancies [23].

## 4. Immune Checkpoint Blockade in Malignancies

Malignancies with a high gene mutational load putatively generate more neo-antigens that may be targeted by the immune system, so that they are more immunogenic. Hence, an important requisite for the survival of these malignant cells is to escape immunosurveillance, in many instances by expression of ligands for immune checkpoint proteins on immune cells [9,11]. For these immunogenic malignancies that depend on escape from immunosurveillance for their proliferation, ICI constitutes a potential therapy. In solid malignancies, ICI is less effective, related to difficult access of immune cells to the tumors, and a more immunosuppressive microenvironment [9,11]. On the other hand, ICI is most successful in selected hematological malignancies, owing often to easy access of immune cells to the tumors, and a microenvironment conducive to immunologic mechanisms [9,11]. The immunologic mechanisms and pathobiologic bases underpinning these clinical responses and differences have been discussed in details elsewhere [9,11].

## 5. Immune Checkpoint Blockade in Lymphomas

### 5.1. cHL

The prototype of lymphoma showing a remarkable response to immune checkpoint inhibition is cHL. Pathologically, cHL is characterized by Hodgkin Reed Sternberg (HRS) cells scattered in a background of inflammatory cells, comprising CD8+ and CD4+ T-cells, and Tregs. These reactive immune cells have an exhausted phenotype with loss of effector function. This is owing to over-expression of PDL-1 and PDL-2 on the malignant HRS cells, caused by amplification of the 9p24.1 locus. Furthermore, the 9p24.1 amplicon includes *JAK2*, and activation of the JAK/STAT signaling pathway further drives *PD-L1* transcription [24]. Binding of PD-L1 and PD-L2 on RHS cells to PD-1 on the infiltrating T-cells leads to suppression of effector T-cell function.

The efficacy of single-agent PD-1 blockade was initially tested in R/R patients, who had failed or were ineligible for autologous hematopoietic stem cell transplantation (AHSCT) (Table 1). In the phase II CheckMate-205 study, 243 patients with R/R disease that had failed AHSCT were treated with nivolumab (3 mg/kg every 2 weeks) until disease progression or unacceptable toxicity. The overall response rate (ORR) was 69% with a complete response (CR) rate of 16%. Median time to response was 2 months, and median duration of response (DOR) was 16.6 months [25]. In the phase II KEYNOTE-087 study, 210 patients who had failed at least two lines of therapy including AHSCT and/or brentuximab vedotin (BV) were treated with pembrolizumab (200 mg once every 3 weeks) until disease progression or unacceptable toxicity. The ORR was 69% with a CR rate of 22.4% [26]. Long-term follow-up data from the KEYNOTE-087 and CheckMate-205 trials showed durable responses, with ORR of >70% and favorable overall survival (OS) [27,28]. The phase III KEYNOTE-204 study enrolled patients who had failed or were ineligible for AHSCT. Patients were randomized to receive pembrolizumab (200 mg every 3 weeks) (N = 151) or BV (1.8 mg/kg every 3 weeks) (N = 153). The results showed significantly superior progression-free survival (PFS) with pembrolizumab as compared with BV (13.2 months *versus* 8.3 months: *p* = 0.0027) [29].

Combination of PD-1 inhibitors with chemotherapy was next tested (Table 2). The largest study was the SWOG 1826 study, which randomized newly diagnosed patients with advanced stage cHL to either nivolumab-adriamycin, vinblastine and dacarbazine (N-AVD), or BV-AVD. Results showed a significantly superior 2-year PFS of N-AVD at 92% as compared with BV-AVD at 83% [51]. The result has established N-AVD as the new standard of care for patients with advanced-stage cHL fit for anthracycline-based combination therapy. Other combinations, including pembrolizumab + gemcitabine, vinorelbine, and liposomal doxorubicin [52], pembrolizumab + ifosfamide, carboplatin, and etoposide (ICE) [53] and nivolumab + ICE [54] have all shown good responses (ORR: 97.3–100%; CR: 86.5–95%) as first salvage therapy for R/R cHL. These efficacy rates are much higher than historical controls using chemotherapy-only regimens.

The effectiveness of a chemotherapy-free salvage treatment using BV combined with PD-1 blockade in R/R disease has also been studied. In a phase I/II study, nivolumab + BV as second-line therapy for R/R disease led to an ORR of 85% (CR: 67%), with a 3-year PFS of 77% [55]. An ongoing trial (NCT04561206) of R/R cHL examines 4 cycles of BV-nivolumab as induction therapy, followed by an additional 12 cycles if CR is achieved. The objective is to test if AHSCT can be omitted in R/R cHL in the era of targeted therapy.

Low-dose anti-PD1 has been explored to economize on drug cost and decrease adverse events (AEs) [89]. A recent systematic review assessed the safety and efficacy of low-dose nivolumab and pembrolizumab in R/R cHL [90]. Low-dose nivolumab was most commonly administered at 40 mg per dose, though some studies used 100–140 mg fixed doses or 0.5 mg/kg. For pembrolizumab, low-dose regimens included a 100 mg fixed dose or a weight-based banding approach: 100 mg for patients 50 ± 10 kg, 2 mg/kg for patients >60 kg, and 200 mg for patients 90 ± 10 kg. Across 10 studies involving 161 patients, ORR was 66–100% with CR at 38–75%, comparable to standard-dose data. Survival outcomes were likewise similar. AEs occurred in 27–93% of cases but were mainly grade 1–2. These findings suggest low-dose anti-PD-1 may represent an effective, affordable, and safe option, particularly in resource-constrained settings.

### 5.2. Primary Mediastinal Large B-Cell Lymphoma (PMBCL)

PMBCL is a distinct subtype of mature aggressive large B-cell lymphoma that predominantly affects young adults with a female preponderance. Despite its aggressive nature, PMBCL patients generally have a favorable prognosis with 5-year survival rates exceeding 80%, especially when early remission is achieved through first-line therapy. However, approximately 20% of patients fail first-line treatment, and more than half of them will be refractory to second-line chemotherapy [5]. PMBCL, similar to cHL, is characterized by frequent genomic alterations at chromosome 9p24.1, including both copy gains and translocations, resulting in overexpression of PD-L1 and PD-L2 [91]. This provides the biological basis for the use of PD-1 blockade in PMBCL.

The phase Ib KEYNOTE-013 study and phase II KEYNOTE-170 trial collectively enrolled 74 patients with heavily pre-treated disease (median of three previous treatment lines). In KEYNOTE-013, pembrolizumab achieved an ORR of 48% (CR: 33%). KEYNOTE-170 showed consistent results, with an ORR of 45% (CR: 13%) [30]. At a median follow-up of 48.7 months, the ORR remained at 41.5% (CR: 20.8%), with a median DOR not reached. None of the patients who achieved CR had disease progression at data cutoff. The 4-year PFS was 33.0%, and the 4-year OS was 45.3% [92]. These results led to approval of pembrolizumab for patients with R/R PMBCL [30].

The phase I/II CheckMate 436 study evaluated the combination of nivolumab with BV in 30 patients with R/R PMBCL, which showed an ORR of 70% (CR: 43%). Median DOR, PFS, and OS were not reached at a median follow-up of 11.1 months [56]. A 3-year follow-up confirmed durability of responses, with eleven responding patients consolidated with AHSCT or allogeneic HSCT, resulting in a 2-year CR of 80–100% [93]. A single-center real-world study of 46 patients treated with pembrolizumab monotherapy (N = 31) or nivolumab + BV (N = 15) showed an ORR of 48.6% (CR: 40.5%). With a median follow-up of 5 years, the median OS was not reached, and PFS was 47.1% at 8 years. Among the study population, nine patients received ICIs as bridging therapy to chimeric antigen receptor (CAR)-T cell therapy, achieving CR in 5 patients. Three patients originally planned to receive CAR-T cell therapy with ICIs as bridging achieved CR and were reassigned to maintenance ICI, and had remained in CR after 35–36 cycles [57].

### 5.3. NK/T-Cell Lymphoma (NKTCL)

NKTCL is a rare, aggressive lymphoma with a predilection for Asian and South American populations, characterized by universal EBV infection in the lymphoma cells and expression of the multidrug resistance phenotype. For patients with R/R disease, conventional salvage therapies have shown poor outcomes, indicating that novel therapeutic approaches are needed.

Owing to EBV infection, NKTCL has unique pathobiologic characteristics. There is upregulation of PD-L1 expression and *PD-L1*/*PD-L2* gene alterations in most cases. Furthermore, EBV LMP1 activates the NF-κB pathway, leading to production of cytokines including tumor necrosis factor-alpha and IFN-γ. These cytokines lead to the upregulation of PD-1 and hence an exhausted phenotype in T-cells in the lymphoma milieu. Therefore, the PD-1/PD-L1 pathway is a potential therapeutic target for NKTCL [15,16].

This approach was first explored in a study evaluating pembrolizumab in seven patients with R/R NKTCL who had failed asparaginase-based regimens and allogeneic HSCT. The ORR was 100%, with five patients attaining CR after a median of seven cycles [31]. Subsequent studies confirmed the efficacy of pembrolizumab in R/R NKTCL [32]. Lower doses of ICIs have been shown to be efficacious, with a retrospective study reporting four of seven patients with R/R NKTCL responding to pembrolizumab administered at doses of 100 mg every 3 weeks, achieving an ORR of 57% (CR, N = 2; PR, N = 2) [33]. Nivolumab has also been shown to be effective in R/R NKTCL even at low doses (40 mg every 2 weeks), with three treated patients showing response [34]. Sintilimab, another PD-1 antibody, has been shown to be effective in the prospective phase II trial ORIENT-4. Among 28 patients with R/R NKTCL, ORR was 75% (CR: 21.4%); and at a median follow-up of 30.4 months, the median OS was not reached [35]. Tislelizumab, a PD-1 inhibitor with unique engineering to avoid Fc gamma receptor binding, had been studied in a multicentre phase II study in patients with NKTCL. Most had advanced (stage III/IV: 63.6%) and refractory (45.5%) disease. The ORR was 31.8% (CR: 18.2%), with a median DOR not reached at 12.5 months. Avelumab, a PD-L1 inhibitor, had been studied in a phase II open-label single-arm trial in twenty-one patients with R/R ENKTL. The study demonstrated promising single-agent activity, with an ORR of 38% (CR: 24%); and acceptable safety, with most AEs being grade 1–2 and no grade 4 events. High PD-L1 expression on tumor tissue was significantly associated with response (*p* = 0.001), with all CR patients having tumors highly expressing PD-L1 [37].

Combination strategies of ICIs have subsequently been tested. A meta-analysis reported the efficacy of PD-1 and PD-L1 inhibitors in patients with newly-diagnosed or relapsed/refractory NKTCL. Thirteen single-arm clinical studies from 2021 to 2024 were included, with a total of 460 patients. Pooled ORR was 62%, with 1-year OS of 67% and 1-year PFS of 66%. Among the studies included, four studies utilized combination regimens of ICI and chemotherapy, with ORR of 82% and 1-year PFS of 74%; and three studies used ICI and histone deacetylase inhibitors (HDACi), with ORR of 77% and 1-year PFS of 91%. Combination regimens outperformed monotherapy, particularly in R/R patients (ORR 79% *versus* 47%) [58].

The phase Ib/II SCENT trial investigated sintilimab plus the HDACi chidamide in patients with R/R NKTCL, demonstrating an ORR of 59.5% (CR: 48.6%). Notably, this combination achieved a median DOR of 25.3 months and a median PFS of 23.2 months, representing a significant improvement over monotherapy approaches [59]. The proposed mechanism involves the epigenetic immunomodulatory effects of chidamide in increasing HLA class I expression, thereby enhancing antigen presentation and therefore tumor immunogenicity, while simultaneously reducing immunosuppressive cell populations. These synergistic effects may restore effector T-cell function even in cases previously refractory to anti-PD-1 monotherapy [94].

Combination of PD-1 inhibitors with asparaginase-based chemotherapy leverages both metabolic targeting and immune checkpoint blockade. A phase II study SPIRIT evaluated sintilimab combined with P-GEMOX (pegaspargase, gemcitabine, oxaliplatin) in treatment-naive advanced NKTCL, achieving remarkable results with 100% ORR (CR: 85%). The 24-month PFS was 64% with a 36-month OS of 76%, establishing this as a highly effective first-line regimen [60]. A chemotherapy-free regimen has been attempted, with a phase II study investigating pegaspargase plus sintilimab in newly diagnosed advanced NKTCL. The ORR was 68% (CR: 59%), with excellent 2-year survival rates (PFS: 68%; OS 86%) [61].

Combination of the PD-1 inhibitor cemiplimab with the anti-CD38 antibody isatuximab has been evaluated. Resistance to PD-1 inhibitors may result from CD38 upregulation, which suppresses CD8+ T-cell activity and promotes Treg-mediated immunosuppression. By targeting CD38, isatuximab may mitigate these effects and enhance PD-1 blockade efficacy, particularly as up to 50% of NKTCLs express CD38. A phase 2 single-arm trial evaluated this combination in thirty-seven patients with R/R NKTCL, resulting in an ORR of 65% (CR: 51%). Median PFS was 9.5 months, and OS was not reached after a follow up of 30 months. Responders often had PD-L1 3′-UTR disruptions or high PD-L1 expression. Toxicities were mostly grade 1–2, with grade ≥3 events in 32% and no treatment-related deaths [62]. The combination showed durable responses and manageable safety, supporting potential synergy between PD-1 and CD38 blockade.

### 5.4. Primary Large B-Cell Lymphoma of Immune-Privileged Sites (IP-LBCL)

Lymphomas of immune-privileged sites are rare aggressive B-cell lymphomas that occur in anatomical sanctuary sites with limited immune surveillance. They include primary central nervous system lymphoma (PCNSL), primary testicular lymphoma (PTL) and primary vitreoretinal large B-cell lymphoma (PVRL). PCNSL and PTL show amplification or copy number alterations at 9p24.1 and hence the *PD-L1* and *PD-L2* genes in nearly 50% of cases. In EBV-positive PCNSL, as in other EBV-associated lymphoid malignancies, viral infection further contributes to overexpression of PD-L1 and PD-L2 [95], fostering an immunosuppressive tumor microenvironment that results in immune escape. The PD-1 pathway therefore represents a valid therapeutic target.

Clinical studies have shown promising results for PD-1 blockade in patients with R/R PCNSL and PTL. An early study of five patients (PCNSL, N = 4; PTL, N = 1) treated with nivolumab 3 mg/kg every 2 weeks showed that all patients achieved an objective response (CR, N = 4; PR, N = 1), with 3 patients having ongoing remission for over a year [38]. Another retrospective analysis of 22 patients with R/R PCNSL treated with nivolumab showed an ORR of 41% (CR, N = 6; PR, N = 3). The median DOR was impressive at 20.9 months [39]. A systematic review and meta-analysis of seven studies involving 127 patients (PCNSL, N = 124; PTL: N = 3) treated with nivolumab or pembrolizumab showed a pooled ORR of 67.1% (CR: 42.8%; PR: 17.1%) and a pooled 6-month PFS of 34.8%. For PCNSL, the pooled ORR was 61.8% (CR: 40.7%). These responses compared favorably to other salvage therapies in this challenging patient population. Importantly, 60.6% of patients analyzed had previously received high-dose methotrexate-based chemotherapy, confirming the utility of ICIs in heavily pretreated patients [40].

A phase I/Ib study evaluated high-dose methotrexate-containing induction chemotherapy followed by nivolumab consolidation in older patients (≥65 years) with previously untreated PCNSL who were unsuitable for whole-brain radiotherapy. Patients received at least three cycles of high-dose methotrexate followed by nivolumab consolidation for six cycles. Patients who received at least one cycle of nivolumab consolidation achieved an ORR of 83% (CR: 75%), with 1-year OS and PFS of 91.7% and 66.1%, respectively [41]. This approach implies that ICI may be a potentially less toxic alternative to maintain remission for older patients.

Despite the promising efficacy of ICIs in IP-LBCL, primary and acquired resistance remain significant challenges. The loss of HLA class I and II expression is particularly frequent in PTL, and may limit the effectiveness of T-cell-mediated therapies, highlighting the need for combination approaches or alternative immune activation strategies. The SMTR (sintilimab, high-dose methotrexate, temozolomide, rituximab) regimen used as frontline treatment of PCNSL in a phase II study achieved a very high ORR of 96.3% (CR: 92.6%), with manageable immune-related toxicities [63]. In R/R PCNSL, ibrutinib + nivolumab demonstrated an ORR of 78% (CR: 50%) with some durable remissions [64], highlighting biological synergy between BTK inhibition and PD-1 blockade. Therefore, the adjunctive use of ICIs in the treatment of PCNSL should be actively explored.

### 5.5. Mycosis Fungoides/Sézary Syndrome

Mycosis fungoides (MF) and Sézary syndrome (SS) are malignancies of skin-homing CD4+ cells, which constitute the majority of cutaneous T-cell lymphomas (CTCL). While early-stage disease is often managed effectively with skin-directed therapies, advanced-stage (III/IV) MF/SS is currently incurable and associated with significant morbidity and mortality. Systemic therapies, including histone deacetylase inhibitors, retinoids, and the anti-CCR4 antibody mogamulizumab, provide transient benefits, but responses are often not durable, and the prognosis remains poor [96].

Malignant cutaneous T cells in MF and SS express PD-1 and CTLA-4. PD-L1 is often upregulated on macrophages and dendritic cells in the tumor microenvironment, in response to interferon-gamma produced by the malignant T cells [97]. This produces a state of adaptive immune resistance and provides the biological basis for the use of PD-1 inhibitors.

Most studies on the use of ICIs in MF/SS were single-arm, phase I/II trials and case series. A multicentre phase II trial studied pembrolizumab in 24 patients with advanced, heavily pre-treated MF/SS. The ORR was 38% (CR: 8%; PR: 29%), with responses durable and a median DOR not reached at 58 weeks of follow-up. About half of the patients with SS experienced cutaneous flare reactions, found to be associated with high PD-1 expression on Sézary cells [42]. Nivolumab demonstrated modest activity in a phase I study, achieving an ORR of 15% in 13 MF patients, with median PFS of 10 weeks [43]. Tislelizumab showed promising results in a phase II study with a cohort of 11 CTCL patients (MF: 8; SS: 3), achieving an ORR of 45.5% (CR: 9.1%). Median DOR was 11.3 months with a median PFS of 16.8 months [36].

Combination approaches have been studied. In a randomized phase II trial, the anti-PD-L1 antibody durvalumab plus lenalidomide achieved an ORR of 75%, which was superior to durvalumab monotherapy (ORR: 42%) [65]. Preliminary data from a phase Ib study showed encouraging responses of a combination of pembrolizumab with the dihydrofolate reductase inhibitor pralatrexate and decitabine in heavily pre-treated patients with CTCL [66], suggesting that the integration of pembrolizumab into an epigenetic targeted backbone was safe and might enhance outcomes.

Very rarely, patients with T-cell lymphomas including CTCL who received ICI might developed a “hyperprogressive” disease. A proposed mechanism is that PD-1 exerts a tumor-suppressive effect on malignant T cells with constitutive TCR activation, and blockade of PD-1 may suppress its inhibitory role, accelerating the progression of CTCL [98].

Hence, ICIs represent a promising but still evolving therapeutic option for R/R MF/SS. Careful patient selection, biomarker development, and safety monitoring remain critical for optimizing outcomes in this challenging patient population.

## 6. Immune Checkpoint Blockade in Other Hematological Malignancies

### 6.1. Multiple Myeloma

Multiple myeloma (MM) remains incurable in the majority of patients, despite advances in immunomodulatory drugs, proteasome inhibitors, anti-CD38 antibodies, and anti-BCMA therapies, prompting investigations of the efficacy of ICI.

KEYNOTE-013 was a phase Ib study of single-agent pembrolizumab in patients with hematological malignancies including R/R MM. In 30 enrolled patients, only stable disease (SD) was achieved in 17 cases (56.7%), at a median follow-up of 19.9 months [44]. A phase Ib study of nivolumab in patients with R/R hematological malignancies included 27 patients with R/R MM. The best response was SD in 17 (63%) patients, lasting a median of 11.4 weeks [64]. A combination study of ipilimumab and nivolumab was conducted in 65 hematological patients, among whom 7 had MM. Outcome was unimpressive, with four patients dying and a median PFS of merely 2.2 months [67]. Overall, single agent ICIs were not associated with meaningful response.

Pre-clinical evidence suggests that PD-1/PD-L1 signaling in myeloma cells may induce resistance to bortezomib and melphalan, so combinatorial strategies of ICIs and proteasome inhibitors have been studied. In preclinical studies, lenalidomide has been shown to be synergistic with PD-1 blockade by enhancing the effect of T- and NK-cell-mediated cytotoxicity, and downgrading PD-L1 on plasma cells [99]. The KEYNOTE-023 trial tested pembrolizumab with carfilzomib and low-dose dexamethasone in R/R MM. Among 10 patients, the ORR was 70%, with median PFS and OS at 14.3 and 22.5 months [68]. The study also tested pembrolizumab in combination with lenalidomide and low-dose dexamethasone in sixty-six patients, resulting in an ORR of 44%, a median PFS of 7.2 months and an OS not reached [69]. However, further phase III trials were abandoned due to a higher mortality and serious AEs in the PD-1 arms, including myocarditis, infections, and intestinal ischemia [100].

Preclinical studies suggest synergy between daratumumab and ICIs, as daratumumab depletes CD38+ immunosuppressive cells, with PD-1 inhibition potentially enhancing T-cell activity and reversing immune suppression [99]. In a phase II study, nivolumab combined with daratumumab achieved an ORR of 50%. In daratumumab-resistant patients, daratumumab-durvalumab showed minimal efficacy [70], whereas daratumumab plus atezolizumab with lenalidomide or pomalidomide achieved very good partial response or better in 43–67% of patients [71].

Currently, the role of ICIs in MM remains uncertain. Results with monotherapy have been poor, while combination approaches initially appeared promising. However, excessive and unpredictable toxicities precluded the conduction of phase III studies. Therefore, more studies are needed to clarify the best role of ICIs in the management of MM, and to guide the development of individualized treatment approaches.

### 6.2. Myeloid Malignancies

In contrast to lymphoid malignancies, results of ICI in myeloid malignancies have been less encouraging. Initial results of CTLA4 blockade with ipilimumab showed limited activity in AML, and only in the setting of R/R AML after allo-HSCT, with 5 of 12 patients responding in a phase I/Ib study [45]. However, in patients with MDS after failure of hypomethylating agents (HMA), ipilimumab yielded minimal benefit, with only 1/29 patients achieving a CR, possibly due to lower tolerated dosing [46].

HMAs have been tested in combination with anti-PD-1 antibodies, to explore a potential synergistic interaction. In a phase II study, pembrolizumab was combined with azacitidine in patients with newly diagnosed and R/R AML. The ORR for the entire cohort was 55%, with the best response achieved in newly-diagnosed patients (ORR: 94%; CR/CR with incomplete count recover, CRi: 47%) [72]. In another study involving only ten patients with R/R AML, pembrolizumab with decitabine achieved an ORR of 60% [73]. In a subsequent phase II study, pembrolizumab combined with chemotherapy (high-dose cytarabine) achieved an ORR of 46% (CR/CRi: 38%) [74]. Finally, single-agent pembrolizumab proved ineffective in MDS patients refractory to HMA in the KEYNOTE-013 study, with no cases of CR/PR achieved [47]. Therefore, ICIs are generally ineffective in R/R AML/MDS, and of limited activity combined with HMA and chemotherapy. Resistance is thought to be related to reduced HLA expression, increased expression of alternative immune checkpoints, and an unfavorable T-cell to MDS/AML cell ratio [101].

TIM-3 has been tested as another target, given its expression on leukemic blasts and stem cells. A phase II study of the anti-TIM-3 antibody sabatolimab with HMAs in high-risk MDS and AML showed encouraging ORRs (MDS: 56.9%; AML: 40%), with durable remissions even in patients with adverse-risk mutations in *TP53*, *RUNX1*, and *ASXL1* [75]. However, in the STIMULUS-MDS1 phase II trial, sabatolimab plus HMA did not significantly improve CR or PFS compared with placebo plus HMA [76]. Similarly, the phase III STIMULUS-MDS2 trial failed to meet its primary endpoint of OS, when sabatolimab plus azacitidine was compared with azacitidine [102].

Blockade of CD47 has similarly been explored. The anti-CD47 antibody magrolimab showed modest single-agent activity and putative synergy with azacitidine in a phase Ib trial AML-196, with an ORR of 65% (CR/CRi: 56%) in AML patients; and an ORR of 71% (CR/CRi: 67%) in AML patients with *TP53* mutations [77]. The ENHANCE-2 study was a phase 3 trial that further evaluated the efficacy of magrolimab in *TP53*-mutated AML. It compared magrolimab plus azacitidine *versus* physician’s choice (venetoclax plus azacitidine in the non-intensive arm or standard chemotherapy in the intensive arm) in patients with previously untreated *TP53*-mutated AML. At interim analysis, the OS hazard ratio was 1.19, meeting the definition for futility and resulting in early study termination. In the final analysis, magrolimab + azacitidine, compared with control, showed similar median OS in the non-intensive arm (4.4 months *versus* 6.6 months, *p* = 0.50) and the intensive arm (7.3 months *versus* 11.1 months, *p* = 0.38) [103]. The phase 3 ENHANCE-3 trial evaluated the addition of magrolimab to non-intensive therapy in previously untreated AML patients ineligible for intensive chemotherapy. The trial was stopped early at a prespecified interim analysis due to futility. Among 378 patients, median OS was 10.7 months in the magrolimab plus venetoclax and azacitidine arm *versus* 14.1 months in the placebo plus venetoclax and azacitidine arm (hazard ratio, HR: 1.178; 95% confidence interval, CI: 0.848–1.637); and CR within 6 cycles was 41.3% (magrolimab arm) *versus* 46% (control arm) (odds ratio: 0.856; 95% CI: 0.560–1.307). The addition of magrolimab resulted in significantly more fatal AEs, driven by grade 5 infections and respiratory events. The study demonstrated that adding magrolimab to venetoclax and azacitidine did not improve outcome and was associated with increased toxicity in this population [78].

Evorpacept, a next-generation anti-CD47 antibody with reduced off-tumor toxicity, is now being evaluated in the ASPEN trial for AML/MDS [88].

## 7. Immune Checkpoint Blockade in the Context of Allo-HSCT

The use of ICIs in the setting of allo-HSCT presents unique challenges and opportunities. Pre-transplantation ICI may result in persistence of residual drug post-transplantation, thereby increasing the risk of severe graft-*versus*-host disease (GVHD), attributed to enhanced alloreactive T-cell proliferation but reduced Tregs.

Despite these risks, the use of ICIs before allo-HSCT has been explored to enhance a putative graft-*versus*-tumor (GVT) effect for reduction in disease relapse. In patients with R/R cHL undergoing allo-HSCT, pre-transplantation anti-PD1 antibody therapy showed OS superior to conventional chemotherapy [104], attributed to a graft-*versus*-lymphoma effect. In the KEYNOTE-170 trial, five patients with R/R PMBCL received pembrolizumab before allo-HSCT, resulting in remission in three patients [92]. In the CheckMate-436 study, six R/R PMBCL patients received nivolumab plus BV before allo-HSCT, with 2-year CR rates of 80% [93], suggesting anti-PD1 antibody therapy may be an effective bridge to allo-HSCT.

However, risks of GVHD still constitute a significant barrier in ICI therapy before allo-HSCT. Large multicenter cohorts reported grade 2–4 acute GVHD (aGVHD) of 31–44% by day 100, with grade 3–4 aGVHD of 13–23% in patients with cHL who underwent allo-HSCT after PD-1 blockade [105,106]. The washout interval significantly affected outcomes, with a 42% rate of severe aGVHD when the last anti-PD-1 dose was within 30 days of allo-HSCT. This rate decreased notably after 30 days, with no severe aGVHD observed when there was a gap of more than 60 days before allo-HSCT [107]. The risk of aGVHD caused by PD-1 blockade was reported to be mitigated by post-transplantation cyclophosphamide (PTCy), which resulted in improved OS, and fewer grade 2 to 4 aGVHD and chronic GVHD, in R/R cHL patients with anti-PD1 antibody exposure prior to allo-HSCT [106].

For myeloid malignancies, a prospective study evaluated 27 patients with R/R AML treated with tislelizumab, HMA, and chemotherapy. Among eleven patients undergoing allo-HSCT, 45.5% experienced aGVHD [108]. A follow-up study of fifteen patients showed a 2-year OS of 54% and GVHD-free/relapse-free survival of 48.6% at a median follow-up of 20.9 months. Among haploidentical patients, four received GVHD prophylaxis with anti-thymocyte globulin and reduced-dose PTCy, with no deaths or relapses in this group, suggesting therefore a potential benefit of PTCy [79].

Relapse after allo-HSCT is partly related to loss of GVT and upregulation of PD-1 and CTLA-4 on allogeneic T cells [109], which might be reversed by ICI. In relapsed cHL after allo-HSCT, PD-1 blockade with nivolumab or pembrolizumab resulted in ORR of 77–95% (CR: 50%). These benefits were counter-balanced by GVHD in up to 55% of patients, often steroid-refractory and in some cases fatal [48,110]. Longer ICI-to-HSCT intervals and PTCy-based prophylaxis similarly reduced GVHD [109]. In myeloid malignancies, outcomes of ICI after allo-HSCT were less favorable, with ORRs of merely 0–21% [49,50]. Combining ICIs with HMAs might improve results and needs to be explored [111].

## 8. AEs of Immune Checkpoint Blockade

Immune-related adverse events (IrAEs) are distinct toxicities associated with ICIs. Their exact mechanisms are not fully understood, but are thought to result from T-cell over-activation and cytokine release [112]. IrAEs can be systemic, such as fatigue or cytokine release syndrome, but more often affect specific organs. Common organ-specific irAEs are cutaneous (rash, pruritus), endocrine (thyroid, pituitary, adrenal, pancreas), gastrointestinal (diarrhea, colitis), pulmonary (pneumonitis), and cardiac (myocarditis, pericarditis). The incidence and severity of irAEs vary by ICI type and combination therapy, with anti-CTLA-4 agents such as ipilimumab more linked to gastrointestinal toxicity and hypophysitis, and PD-1/PD-L1 inhibitors more commonly related to thyroid dysfunction and pneumonitis [113]. Toxicity profile also varies according to diseases and treatment regimens. In cHL and PMBCL, adverse effects included hypothyroidism (occurring in 8–29% of patients on nivolumab), hepatotoxicity (with combination therapies such as ipilimumab + BV causing transaminitis in up to 48% of patients), and pneumonitis (in up to 24% of patients on nivolumab) [114]. Trials in multiple myeloma were marked by severe and unique toxicities when ICIs were combined with lenalidomide/pomalidomide, including two reported cases of fatal myocarditis, which led to the suspension of major phase 3 trials KEYNOTE-183 and KEYNOTE-185. In T-cell lymphomas, pembrolizumab led to unique “skin flare” reactions in Sézary syndrome patients (up to 53%) but did not result in treatment discontinuation in any patient [42].

## 9. Future Directions

### 9.1. PD1 Blockade in Combination with Chimeric Antigen Receptor (CAR)-T Cell Therapy

The efficacy of CAR-T cell therapy is often limited by T-cell exhaustion. Blocking the PD-1/PD-L1 pathway has been shown to restore CAR-T cell function, leading to significant interest in combining CAR-T cell therapy with ICIs. The ZUMA-6 trial aims to evaluate the safety and efficacy of axicabtagene ciloleucel in combination with the anti-PD-L1 antibody atezolizumab for R/R DLBCL. In the Phase I analysis of 12 patients, ORR was 90% (CR: 50%). Significantly, the CAR-T cell expansion was more than twice that reported in the ZUMA-1 study [80]. Although the combination appears promising as an initial therapy, the use of ICI after CAR-T failure has yielded mixed results. While some reported CAR-T cell re-expansion in 83.3% of patients after pembrolizumab administration with evidence of increased activation and reduced exhaustion [115], others found little or no consistent re-expansion [116,117]. Furthermore, other exhaustion markers such as LAG-3 and TIM-3 may play an even stronger role in resistance to CAR-T cell therapy [118], indicating that future research must focus on targeting a broader range of checkpoint molecules and determining the optimal timing for ICI administration.

### 9.2. PD1 Blockade in Combination with Epigenetic Therapy

Epigenetic modulation with HDACi aims to enhance immune checkpoint blockade by increasing tumor immunogenicity through upregulation of antigen-presenting genes and enhanced tumor recognition and killing. The combination of PD-1 inhibitors with the HDACis vorinostat or entinostat has shown high efficacy in treating R/R cHL. A Phase I study using vorinostat plus pembrolizumab achieved an ORR of 93% (CR: 64%) in PD-1-naïve/sensitive patients, and induced an ORR of 56% in patients previously refractory to PD-1 blockade [81]. This synergy was further supported by two Phase II trials of entinostat plus pembrolizumab in cHL [82,83]. Preliminary results of one of these trials showed an ORR of 86% (CR: 45%), with responders including those who had failed prior anti-PD-1 therapy [82]. These findings underscore that HDACis enhance tumor immunogenicity in cHL, effectively rejuvenating the immune response when combined with PD-1 blockade.

The combination of anti-PD-1 with HMA has also been studied in cHL. DNA methylation de novo has been reported to drive T-cell exhaustion and reduce response to PD-1 blockade, so that methylation inhibition with low-dose decitabine is purportedly able to increase tumor immunogenicity, T-cell infiltration and antitumor response. A phase II study enrolled 86 patients with R/R cHL and randomly assigned them to camrelizumab (a fully humanized IgG4/κ anti-PD-1 antibody) monotherapy, or decitabine plus camrelizumab combination therapy. In anti-PD-1 naïve patients, CR rate was 32% with carmelizumab monotherapy *versus* 71% in combination therapy of decitabine plus camrelizumab (*p* = 0.003). Among patients previously treated with anti-PD-1, ORR was 52% (CR: 28%) [119]. After an extended median follow-up of 34.5 months, CR rate was 79% in the decitabine-plus-carmelizumab group *versus* 32% in the camrelizumab group (*p* = 0.001). Responses were durable, with median DOR not reached, and median PFS of 35.0 months in the decitabine-plus-camrelizumab group, as compared with DOR of 12.7 months and median PFS of 15.5 months in the camrelizumab monotherapy group. The addition of decitabine improved PFS across most subgroups, particularly in patients with higher tumor burden or ≥3 previous therapies [84]. A retrospective study analyzed 87 patients with R/R cHL who achieved CR after decitabine-plus-camrelizumab epi-immunotherapy. Median relapse-free survival (RFS) was 4.5 years after a follow-up of 5.3 years. Among 57 patients who discontinued therapy after maintaining CR for 1 year, the 2-year post-cessation RFS rate was 78%, with remissions ongoing in most patients. These results showed that the decitabine-plus-camrelizumab combination could achieve long-term disease control [85]. Combination of the HDACi chidamide with decitabine and camrelizumab (CDP) was studied in 52 patients with R/R cHL who previously received decitabine and camrelizumab therapy. CDP therapy proved highly effective, achieving ORR of 94% (CR: 50%) with a median PFS of 29.4 months. Single-cell RNA sequencing (scRNA-seq) analysis of paired tumor samples showed that the efficacy of the CDP regimen correlated with the depletion of a potentially less immune-sensitive CD30-negative HRS-like cell subpopulation and the activation of diverse CD8+ T-cell clones, suggesting CDP alleviated T-cell exhaustion. The scRNA-seq also highlighted a decrease in immunosuppressive interleukin-21 and T helper cells in responders, indicating the triplet therapy overcame PD-1 resistance by targeting the immunosuppressive tumor microenvironment [86].

### 9.3. PD1 Blockade in Combination with Other Immune Checkpoint Inhibitors

Dual immune checkpoint inhibition may potentially overcome compensatory inhibitory pathways. The therapeutic effect of combining ICIs may result from their complementary actions, with anti-CTLA-4 enhancing T cell priming, and anti-PD-1 boosting T cell effector activity. In cHL, the combination of nivolumab, ipilimumab and BV has shown meaningful activity with manageable safety [87].

Beyond PD-1, novel checkpoints such as LAG-3 and TIGIT are being investigated to address adaptive resistance. LAG-3 is co-expressed with PD-1 on exhausted T cells. The anti-LAG3 antibody relatlimab combined with nivolumab has shown clinical benefit in early studies in lymphomas and myeloma. In the RELATIVITY-022 phase I/IIa study, 106 patients with R/R B-cell malignancies received relatlimab alone or with nivolumab. The combination was well tolerated with no unexpected toxicities. Among PD-1/PD-L1-naïve cHL patients, the ORR was 62% (CR: 19%), with a median PFS of 19 months; but responses were lower in cHL and DLBCL that progressed during previous anti-PD-1/PD-L1 therapy [19]. Overall, nivolumab plus relatlimab showed promising activity in PD-1- naïve cHL. Other ongoing trials include phase I studies of LAG-3 blockade in lymphomas (HLX26, NCT05078593; Sym022, NCT03489369); a phase II study of relatlimab, nivolumab and azacitidine in R/R or newly diagnosed unfit patients with AML (NCT04913922); and a study of relatlimab combined with TIGIT blockade plus pomalidomide/dexamethasone in R/R MM (NCT04150965). Currently, most trials remain early-staged, with limited efficacy data.

## 10. Conclusions

Leveraging the immune system in treating hematological malignancies has significantly improved patient outcome. ICIs have shown favorable efficacy in various hematological malignancies. There is considerable scope to enhance checkpoint-based therapies in diseases not responding favorably to single-agent ICI therapy, including myeloid malignancies and MM. Further developments of ICIs focus on combinations, either with conventional chemotherapy or other targeted agents such as bispecific antibodies, CAR-T cells, and antibody-drug conjugates. Most of these approaches are still in preclinical development. Future efforts will be devoted to identifying biomarkers predictive of response or resistance, optimizing the timing of ICI therapy, and developing innovative combination strategies.

## Figures and Tables

**Table 1:** Single-agent immune checkpoint inhibitors (ICI) in haematological malignancies.

			ORR					
ICI	Disease	Study	CR	PR	DOR	PFS	OS	Reference
Nivolumab	R/R cHL	CheckMate-205 (Phase II trial)	16%	53%	16.6 m	Median: 14.7 m	1-year: 92%	(Armand et al.; 2018, [25])
Pembrolizumab	R/R cHL	KEYNOTE-087 (Phase II trial)	22.4%	46.6%	NR	6 m: 72.4% 9 m: 63.4%	6 m: 99.5% 9 m: 97.5%	(Chen et al.; 2017, [26])
Pembrolizumab	R/R cHL	KEYNOTE-204 (Phase III trial)	25%	41%	20.7 m	Median: 13.2 m	NA	(Kuruvilla et al.; 2021, [29])
Pembrolizumab	R/R PMBCL	KEYNOTE-013 (Phase Ib trial)	33%	15%	NR	Median: 10.4 m 12 m: 47%	Median: 31.4 m 12 m: 65%	(Armand et al.; 2019, [30])
Pembrolizumab	R/R PMBCL	KEYNOTE-170	13%	32%	NR	Median: 5.5 m 12 m: 65%	Median: NR 12 m: 58%	(Armand et al.; 2019, [30])
Pembrolizumab	R/R NKTCL	Retrospective case series	71.4%	28.5%	NR	NA	NA	(Kwong et al.; 2017, [31])
Pembrolizumab	R/R NKTCL	Prospective phase II, single arm trial	44%	6%	NA	Median: 10 m	Median: 25 m	(Chan & Tse, 2023, [32])
Pembrolizumab (low dose: 100 mg q3w)	R/R NKTCL	Retrospective case series	28.6%	28.6%	4.1 m	4.8 m	5 m	(Li et al.; 2018, [33])
Nivolumab (low dose: 40 mg q2w)	R/R NKTCL	Retrospective case series	66.6%	33.3%	NA	NA	NA	(Chan et al.; 2017, [34])
Sintilimab	R/R NKTCL	ORIENT-4 (Phase II trial)	21.4%	53.6%	4.1 m	NA	Median: NR 24 m: 78.6%	(Tao et al.; 2021, [35])
Tislelizumab	R/R NKTCL	NCT03493451 (Phase II multicenter multicohort trial)	18.2%	13.6%	NA	Median: 2.7 m 1-year: 22.7%	Median: 8.8 m 18 m: 46.4%	(Bachy et al.; 2023, [36])
Avelumab	R/R NKTCL	NCT03439501 (Phase II trial)	24%	14%	NA	Median: 2.7 m	NR	(Kim et al.; 2020, [37])
Nivolumab	R/R PCNSL and PTL	Case series	80%	20%	NA	13–17 m	NR	(Nayak et al.; 2017, [38])
Nivolumab	R/R PCNSL	Nationwide retrospective study	27.2%	13.6%	20.9 m	Median: 2.1 m 1-year: 36.9%	Median: 18.9 m 1-year: 53.1%	(Yi et al.; 2025, [39])
Nivolumab or Pembrolizumab	Relapsed PCNSL and PTL	Systematic review and meta-analysis	42.8%	17.1%	NA	6 m: 34.8%	NA	(Uawithya et al.; 2025, [40])
Nivolumab	PCNSL unsuitable for ASCT/WBI	NCT04022980 (Phase I/IB single-arm multicenter trial)	75%	8%	NA	1-year: 66.1%	1-year: 91.7%	(Park et al.; 2023, [41])
Pembrolizumab	R/R MF and Sézary syndrome	CITN-10 (Phase II trial)	8.33%	29.2%	NR	1-year: 65%	1-year: 95%	(Khodadoust et al.; 2020, [42])
Nivolumab	R/R hematological malignancies (MF: 13)	NCT01592370 (Phase I trial)	0%	15%	NA	Median: 10 wk	NA	(Lesokhin et al.; 2016, [43])
Tislelizumab	R/R T- and NK-cell lymphomas (CTCL: 11)	NCT03493451 (Phase II trial)	9.1%	36.4%	11.3 m	Median: 16.8 m	Median: NR	(Bachy et al.; 2023, [36])
Pembrolizumab	R/R MM	KEYNOTE-013 (Phase Ib trial)	0%	0%	NA	Median: 2.7 m	Median: 20.2 m	(Ribrag et al.; 2019, [44])
Nivolumab	R/R hematological malignancies (MM: 27)	NCT01592370 (Phase Ib trial)	4%	0%	NA	Median: 10 wk	NA	(Lesokhin et al.; 2016, [43])
Ipilimumab	R/R hematological malignancies after allo-HSCT	NCT01822509 (Phase I/Ib trial)	23%	9%	NR	NA	1-year: 49%	(Davids et al.; 2016, [45])
Ipilimumab	Higher risk MDS failing HMAs	Phase Ib trial	3.4%	0%	3 m	NA	NA	(Zeidan et al.; 2018, [46])
Pembrolizumab	MDS failing HMA	KEYNOTE-013 (Phase Ib trial)	0%	0%	NA	NA	Median: 6 m 2-year: 17%	(Garcia-Manero et al.; 2022, [47])
Nivolumab or Pembrolizumab	Relapsed lymphomas (cHL, FL) after allo-HSCT	Multicentre retrospective analysis	50%	26.7%	NA	NA	NA	(Haverkos et al.; 2017, [48])
Pembrolizumab	R/R hematological malignancies post allo-HSCT (AML, MDS, cHL, DLBCL)	Prospective study	22%	0%	NA	Median: 2.9 m	Median: 23.3 m	(Godfrey et al.; 2023, [49])
Nivolumab	R/R hematological malignancies post allo-HSCT	NCT01822509 (Phase 1 trial)	NA	NA	NA	1-year: 23%	1-year: 56%	(Davids et al.; 2020, [50])
HLX26 (anti-LAG3 antibody)	Advanced or metastatic solid tumors or lymphomas	NCT05078593 (Phase I study)	NA	NA	NA	NA	NA	(Liu et al.; 2023, [18])

ORR: overall response rate; CR: complete response; PR: partial response; DOR: duration of response (median); PFS: progression free survival; OS: overall survival; R/R: relapsed/refractory; cHL: classical Hodgkin lymphoma; PMBCL: primary mediastinal B-cell lymphoma; NKTCL: natural killer/T-cell lymphoma; PCNSL: primary central nervous system lymphoma; PTL: primary testicular lymphoma; MF: mycosis fungoides; MM: multiple myeloma; CTCL: cutaneous T-cell lymphoma; MDS: myelodysplastic neoplasm; FL: follicular lymphoma; AML: acute myeloid leukemia; DLBCL: diffuse large B-cell lymphoma; HMA: hypomethylating agent; ASCT: autologous stem cell transplantation; WBI: whole brain irradiation; allo-HSCT: allogeneic hematopoietic stem cell transplantation; m: months; wk: weeks; NA: not available; NR: not reached.

**Table 2:** Combination therapy involving immune checkpoint inhibitors (ICI) in haematological malignancies.

				ORR					
ICI	Other Agent(s)	Disease	Study	CR	PR	DOR	PFS	OS	Reference
Nivolumab	Adriamycin, vinblastine, dacarbazine	Newly diagnosed advanced-stage cHL	SWOG 1826 (Phase III trial)	NA	NA	NA	2-year: 92%	2-year: 99%	(Herrera et al.; 2024, [51])
Pembrolizumab	Gemcitabine, vinorelbine, liposomal doxorubicin	R/R cHL	NCT03618550 trial (Phase II trial)	95%	5%	NA	NR	NR	(Moskowitz et al.; 2021, [52])
Pembrolizumab	Ifosfamide, carboplatin, etoposide (ICE)	R/R cHL	NCT03077828 (Phase II trial)	86.5%	10.8%	NA	2-year: 87.2%	2-year: 95.1%	(Bryan et al.: 2023, [53])
Nivolumab	ICE	R/R cHL	Phase II trial	86%	14%	NA	2-year: 94%	2-year: 100%	(Mei et al.; 2022, [54])
Nivolumab	Brentuximab vedotin	R/R cHL	NCT02572167 (Phase I-II trial)	67%	18%	NA	3-year: 77%	3-year: 93%	(Advani et al.; 2021, [55])
Nivolumab	Brentuximab vedotin	R/R PMBCL	CheckMate 436 (Phase I-II trial)	43%	27%	NR	6 m: 64.5% Median: NR	6 m: 86.3% Median: NR	(Zinzani et al.; 2019, [56])
Nivolumab	Brentuximab vedotin	R/R PMBCL	Monocentric real-life retrospective study	40.5%	8.1%	NA	8-year: 47.1%	Median: NR	(Zinzani et al.; 2024, [57])
PD-1/PD-L1 inhibitors	Chemotherapy, histone deacetylase inhibitors	NKTCL	Meta-analysis	NA	NA	NA	1-year: 66% 2-year: 59%	1-year: 67% 2-year: 47%	(Yang et al.; 2025, [58])
Sintilimab	Chidamide	R/R NKTCL	SCENT (Phase Ib/II trial)	48.6%	10.9%	25.4 m	Median: 23.2 m 3-year: 38.5%	Median: 32.9 m 3-year: 47.4%	(Gao et al.; 2024, [59])
Sintilimab	Pegaspargase, gemcitabine, oxaliplatin	Newly diagnosed advanced stage NKTCL	SPIRIT (Phase II trial)	85%	15%	NA	2-year: 64%	3-year: 76%	(Tian et al.; 2024, [60])
Sintilimab	Pegaspargase	Newly diagnosed advanced stage NKTCL	NCT04096690 (Phase II trial)	59%	9%	NR	2-year: 68%	2-year: 96%	(Xiong et al.; 2024, [61])
Cemiplimab	Isatuximab	R/R NKTCL	NCT04763616 (Phase II trial)	51%	14%	29.4 m	Median: 9.5 m	NR	(Kim et al.; 2025, [62])
Sintilimab	High-dose methotrexate, temozolomide, rituximab	Newly diagnosed PCNSL	ChiCTR1900027433 (Phase II trial)	92.6%	3.7%	NR	NR	NR	(Zeng et al.; 2024, [63])
Nivolumab	Ibrutinib	R/R PCNSL	NCT03770416 (Phase II single-center trial)	50%	27.8%	NA	Median: 6.5 m 1-year: 42%	Median: 21 m 1-year: 63%	(Chihara et al.; 2025, [64])
Durvalumab	Lenalidomide	R/R CTCL	NCT03011814 (Phase II trial)	NA	NA	NA	Median: NR 1-year: 73%	NA	(Querfeld et al.; 2024, [65])
Pembrolizumab	Pralatrexate and/or decitabine	R/R PTCL and CTCL	NCT03240211 (Phase Ib trial)	11.1%	22.2%	NA	NA	NA	(Roberts et al.; 2022, [66])
Nivolumab	Ipilimumab	R/R blood malignancies (MM: 7)	CheckMate 039 (Phase 1b trial)	0%	0%	NA	Median: 2.2 m	Median: 7.6 m	(Ansell et al.; 2016, [67])
Pembrolizumab	Carfilzomib, low-dose dexamethasone	R/R MM	Cohort 2 of KEYNOTE-023 (Phase I trial)	0%	70%	14.1 m	Median: 14.3 m	Median: 22.5 m	(Moreau et al.; 2021, [68])
Pembrolizumab	Lenalidomide, low-dose dexamethasone	R/R MM	KEYNOTE-023 (Phase I trial)	4%	40%	18.7 m	Median: 7.2 m	Median: NR	(Mateos et al.; 2019, [69])
Durvalumab	Daratumumab	refractory MM	NCT03000452 (Phase II trial)	0%	0%	55 d	Median: 30 d	Median: NR	(Frerichs et al.; 2021, [70])
Atezolizumab	Daratumumab (+/− lenalidomide, pomalidomide)	R/R MM	NCT02431208 (Phase Ib trial)	NA	43–67%	NA	NA	NA	(Cho et al.; 2018, [71])
Pembrolizumab	Azacitidine	Cohort 1: R/R AML Cohort 2: newly diagnosed AML	Phase II trial	Cohort 1: 14% Cohort 2: 47%	Cohort 1: 4% Cohort 2: 12%	NA	NA	Median: 13.1 m	(Gojo et al.; 2019, [72])
Pembrolizumab	Decitabine	R/R AML	NCT02996474 (Phase I/II trial)	NA	60%	NA	NA	Median: 10 m	(Goswami et al.; 2022, [73])
Pembrolizumab	High-dose cytarabine	R/R AML	NCT02768792 (Phase II trial)	38%	8%	NA	NA	Median: 11.1 m	(Zeidner et al.; 2021, [74])
Sabatolimab	HMA	Very-high/high-risk MDS Newly diagnosed AML	NCT03066648 (Phase Ib trial)	NA	MDS: 56.9% AML: 40%	MDS: 16.1 m AML: 12.6 m	MDS 1-year: 51.9% AML 1-year: 27.9%	NA	(Brunner et al.; 2021, [75])
Sabatolimab	HMA	Intermediate-, high- and very high-risk MDS	STIMULUS-MDS1 (Phase II trial)	22%	NA	NA	Median: 11.1 m	NA	(Zeidan et al.; 2024, [76])
Sabatolimab	Azacitidine	Higher-risk MDS or CMML-2	STIMULUS-MDS2 (Phase III trial)	19.6%	1.5%	NA	Median: 13.6 m	Median: 22.3 m	(Zeidan et al.; 2024, [76])
Magrolimab	Azacitidine	AML	AML-196 (Phase Ib trial)	56%	3%	NA	NA	*TP53*-WT Median: 18.9 m *TP53*-mutant Median: 12.9 m	(Sallman et al.; 2021, [77])
Magrolimab	Venetoclax and azacitidine	Unfit newly diagnosed AML	ENHANCE-3 study (Phase III trial)	41.3%	NA	NA	NA	Median: 10.7 m	(Daver et al.; 2025, [78])
Tislelizumab	HMA and cytarabine, aclarubicin/idarubicin, granulocyte colony-stimulating factor	R/R AML	NCT04541277 (Phase II study)	NA	NA	NA	2-year: 48.6%	2-year: 54%	(Wang et al.; 2024, [79])
Ateolizumab	Axicabtagene ciloleucal	R/R DLBCL	ZUMA-6 (Phase I trial)	60%	30%	NA	NA	NA	(Jacobson et al.; 2018, [80])
Pembrolizumab	Vorinostat	R/R cHL	Phase I trial	33%	40%	14 m	Median: 14.9 m	Median: NR	(Herrera et al.; 2021, [81])
Pembrolizumab	Entinostat	R/R cHL	Phase II trial	45%	41%	NA	1-year: 72%	NA	(Sermer et al.; 2021, [82])
Pembrolizumab	Entinostat	R/R cHL and FL	Phase II trial	NA	64%	6 m	NA	NA	(Sermer et al.; 2019, [83])
Camrelizumab	Decitabine	R/R cHL	NCT02961101 and NCT03250962 (phase II study)	79%	16%	NR	Median: 35 m 2-year: 67%	NA	(Liu et al.; 2021, [84])
Camrelizumab	Decitabine	R/R cHL	Retrospective analysis	NA	NA	NA	Median: 4.5 years 2-year: 78%	NA	(Wang et al.; 2023, [85])
Camrelizumab	Chidamide, decitabine	R/R cHL	NCT04233294 (Phase II trial)	50%	44%	CR: 33.2 m PR: 20.9 m	Median: 29.4 m	NA	(Nie et al.; 2024, [86])
Nivolumab, ipilimumab	Brentuximab vedotin	R/R cHL	NCT01896999 (Phase I/II trial)	73%	NA	NA	Median: NR	Median: NR	(Diefenbach et al.; 2020, [87])
Nivolumab	Relatlimab	R/R B-cell malignancies	RELATIVITY-022 (Phase I/IIa trial)	cHL: 19%	cHL: 43%	NA	cHL Median: 10 m	NA	(Gopal et al.; 2025, [19])
Evorpacept	Venetoclax, azacitidine	R/R or newly- diagnosed AML with adverse risk genetics and ineligible for intensive induction therapy	ASPEN-05 (Phase I/II trial—ongoing)	NA	NA	NA	NA	NA	(Garcia-Manero et al.; 2022, [88])

ORR: overall response rate; CR: complete response; PR: partial response; DOR: duration of response; PFS: progression free survival; OS: overall survival; cHL: classical Hodgkin lymphoma; NA: not available; NR: not reached; PMBCL: primary mediastinal B cell lymphoma; R/R: relapsed/refractory; NKTCL: natural killer/T-cell lymphoma; m: months; PCNSL: primary central nervous system lymphoma; CTCL: cutaneous T-cell lymphoma; PTCL: peripheral T-cell lymphoma; MM: multiple myeloma; d: days; HMA: hypomethylating agents; CMML: chronic myelomonocytic leukemia; WT: wildtype; DLBCL: diffuse large B-cell lymphoma; FL: follicular lymphoma.

## Data Availability

The authors have no original data to share.
